# LCAT deficiency: a systematic review with the clinical and genetic description of Mexican kindred

**DOI:** 10.1186/s12944-021-01498-6

**Published:** 2021-07-13

**Authors:** Roopa Mehta, Daniel Elías-López, Alexandro J. Martagón, Oscar A Pérez-Méndez, Maria Luisa Ordóñez Sánchez, Yayoi Segura, Maria Teresa Tusié, Carlos A. Aguilar-Salinas

**Affiliations:** 1 Department of Endocrinology and Metabolism, Instituto Nacional de Ciencias Médicas y Nutrición, Salvador Zubirán, Av. Vasco de Quiroga 15, Belisario Domínguez Secc. 16, , Tlalpan 14080 México City, México; 2 Tecnologico de Monterrey, Escuela de Medicina y Ciencias de la Salud, Monterrey, N.L México; 3 Department of Molecular Biology, Instituto Nacional de Cardiología Ignacio Chávez, México City, México; 4 Department of Molecular Biology, Instituto Nacional de Ciencias Médicas y Nutrición, Salvador Zubirán, México City, México

**Keywords:** LCAT deficiency, HDL cholesterol, Ethnicity, Coronary artery disease, Renal disease, Cardiovascular risk

## Abstract

**Background:**

LCAT (lecithin-cholesterol acyltransferase) deficiency is characterized by two distinct phenotypes, familial LCAT deficiency (FLD) and Fish Eye disease (FED). This is the first systematic review evaluating the ethnic distribution of LCAT deficiency, with particular emphasis on Latin America and the discussion of three Mexican-Mestizo probands.

**Methods:**

A systematic review was conducted following the PRISMA (Preferred Reporting Items for Systematic review and Meta-Analysis) Statement in Pubmed and SciELO. Articles which described subjects with LCAT deficiency syndromes and an assessment of the ethnic group to which the subject pertained, were included.

**Results:**

The systematic review revealed 215 cases (154 FLD, 41 FED and 20 unclassified) pertaining to 33 ethnic/racial groups. There was no association between genetic alteration and ethnicity. The mean age of diagnosis was 42 ± 16.5 years, with fish eye disease identified later than familial LCAT deficiency (55 ± 13.8 vs. 41 ± 14.7 years respectively). The prevalence of premature coronary heart disease was significantly greater in FED vs. FLD. In Latin America, 48 cases of LCAT deficiency have been published from six countries (Argentina (1 unclassified), Brazil (38 FLD), Chile (1 FLD), Columbia (1 FLD), Ecuador (1 FLD) and Mexico (4 FLD, 1 FED and 1 unclassified). Of the Mexican probands, one showed a novel LCAT mutation.

**Conclusions:**

The systematic review shows that LCAT deficiency syndromes are clinically and genetically heterogeneous. No association was confirmed between ethnicity and LCAT mutation. There was a significantly greater risk of premature coronary artery disease in fish eye disease compared to familial LCAT deficiency. In FLD, the emphasis should be in preventing both cardiovascular disease and the progression of renal disease, while in FED, cardiovascular risk management should be the priority. The LCAT mutations discussed in this article are the only ones reported in the Mexican- Amerindian population.

**Supplementary Information:**

The online version contains supplementary material available at 10.1186/s12944-021-01498-6.

## Introduction

Lecithin cholesterol acyltransferase (LCAT) is a 67 kDa protein, predominantly expressed in the liver [1]. It circulates in plasma bound to high density lipoproteins (HDL) but can also be found on apolipoprotein B100 containing particles [2–4]. It catalyzes the transfer of an unsaturated fatty acid from lecithin to free cholesterol, producing lysolecithin and cholesteryl ester. This reaction occurs on immature HDL particles in the presence of apolipoprotein A-I (apo A-I), and corresponds to the alpha activity of the LCAT enzyme. When this reaction occurs on low density lipoproteins (LDL) or very low density lipoproteins (VLDL) it is referred to as the beta activity. The net result is the formation of hydrophobic cholesterol ester, which is transferred to the lipoprotein core. In the case of HDL, this allows the conversion of discoidal pre-beta 1 particles to mature spherical alpha forms. In addition, the esterification of cholesterol on HDL increases the concentration gradient for free cholesterol between cell membranes and HDL, thus promoting the removal of cholesterol from cells [1, 2]. Another HDL associated serum enzyme is paraoxonase 1 (PON1), this may play a role in the protection of LDL particles from oxidative stress. Hence, low serum concentrations of HDL may impact susceptibility to vascular disease [3].

LCAT deficiency is a rare autosomal recessive disease [4, 5]. Loss of LCAT function causes decreased maturation of HDL particles and increased HDL levels of unesterified cholesterol and phosphatidylcholine. There is no reliable estimate of the prevalence of the disease; in individuals with very low HDL cholesterol (HDL-C) ranges, the estimated prevalence of LCAT deficiency is between 2 and 9 % [6–8]. The disease is characterized by two distinct phenotypes, familial LCAT deficiency (FLD) and Fish Eye disease (FED). In FLD, both the alpha and beta LCAT activity is lost, leading to extremely low plasma HDL-C (below the 5th percentile for the population), premature corneal opacification, hemolytic anemia, proteinuria and renal failure [9]. In FED, only the alpha LCAT activity is lost, the beta activity is preserved, permitting cholesterol esterification on VLDL and LDL but not on HDL [10]. As a result, the HDL particles contain only 20 % cholesteryl ester, as compared to 75 to 80 % in control HDL. These individuals present with corneal opacities and low HDL-C levels, but are free of the renal consequences seen in complete LCAT deficiency. Calabresi et al., have suggested that FLD and FED are not two distinct syndromes, but the same disease showing differing levels of LCAT activity [11]. Despite distinct cholesterol esterification profiles between FED and FLD, they found that the biochemical phenotype was quite similar; this is further supported by the finding of anemia and renal disease in FED cases.

The clinical phenotype is only apparent in individuals who carry two mutant LCAT alleles [12]. The LCAT gene consists of 6 exons, spans 4,200 base pairs and is located on chromosome 16 (16q22) [13]. Of the reported mutations, the majority are associated with the FLD phenotype, with a significant number remaining unclassified (21.5 %) [14].

Mutations in the *LCAT* gene have been recorded in multiple ethnic and racial groups. However, there is no description of LCAT deficiency in terms of ethnic distribution. In particular, there is sparse knowledge of LCAT deficiency syndromes in Latin America. The greater susceptibility of Hispanics for dyslipidemia (in particular phenotypes with low HDL cholesterol) is a well-documented phenomenon [15]. Hispanic ethnicity results from the admixture of native Americans and Spaniards. The Amerindians have suffered catastrophic events (wars, famines, infections), it is likely that genetic selection processes have occurred in this group influencing findings in present day Hispanics [16].

This article reports the results of the first systematic review conducted to explore the distribution of LCAT disorders, particularly those associated with the Amerindian /Hispanic group. In addition, we describe the biochemical and genetic investigation of three previously unreported Mexican probands and their kindred with LCAT deficiency syndromes.

## Methods for systematic review

A systematic search was conducted following the PRISMA (Preferred Reporting Items for Systematic reviews and Meta-Analyses) Statement in Pubmed, Embase and SciELO. (The PRISMA checklist is shown in supplementary Table 1). The initial PICO question was posed as follows: in patients with LCAT disorders (P), the distribution (I) of FLD compared to FED, (C) and the association with ethnicity (especially among Amerindian/Hispanic population) (O). Articles which described subjects with clinical characteristics compatible with an LCAT deficiency disorder (with or without a mutation in the LCAT gene), and reporting of the ethnic group to which the subject belonged, were considered for analysis. All epidemiological studies which contained the following keywords or MeSH terms were considered: Fish eye disease (FED), Familial Lecithin cholesterol acyl transferase deficiency (FLD), LCAT enzyme deficiency (partial and total), LCAT gene mutation or polymorphism, homozygotes, compound heterozygotes, corneal opacities, corneal clouding, low high density cholesterol levels, anemia, renal failure, atherosclerosis. The search strategy is described in the supplementary materials. Articles written in English, Spanish or Portuguese were included. Data collection was carried out by four investigators, commenced in September 3rd, 2018 and concluded in March 1st, 2020. The investigators took care to avoid double counting of cases by working in pairs; one pair including or excluding cases, and the other pair verifying the decision. All decisions had to be reached unanimously by each pair. In addition, the reference lists of review articles and conference abstracts were also considered. Abstracts were independently assessed to identify eligible research reports. The commonest reasons for ineligibility were, insufficient information regarding either the LCAT gene mutation, clinical characteristics or no mention of ethnicity. This systematic review has been registered in the PROSPERO systematic review register with the ID CRD42021229254. The date of registration was after data extraction was completed. This is not ideal. However, data collection began in 2018, at which point registration was not mandatory. Protocol registration and development are now considered desirable prior to initiating any search strategy in systematic analysis [17].

## Methods for biochemical and genetic analysis of lcat probands

Biochemical measurements.

Fasting serum blood samples were obtained from all three probands. These included full blood count, chemistry, a complete lipid profile, erythrocyte fragility studies (in FLD deficiency subjects), and 24-hour urine collection for determination of microalbuminuria (30–299 mg/g) and creatinine clearance. The lipid parameters were measured inthe Institute’s central laboratory. For total cholesterol (TC), HDL-C, LDL cholesterol (LDL-C), triglycerides and glucose measurements commercial enzymatic methods were used (Beckman Coulter). Apolipoprotein A1 and apolipoprotein B concentrations were measured using nephelometric methods (Beckman Coulter).

Measurement of LCAT activity.

α-LCAT activity was measured in plasma using the Chen and Albers method [18]. Briefly: apoAI/phosphatidilcholine/^3^H-cholesterol complexes were incubated with plasma in a shaking water bath for 1 h al 37 °C (esterification was linear during this time). The reaction was stopped, and lipids were extracted. Esterified and unesterified cholesterol were separated by thin-layer chromatography, and the radioactivity was counted. LCAT specific activity was expressed as the nanograms of cholesterol esterified by 1 mL of plasma in 1 h (nmol/mL/h).

Measurement of Paroxonase-1 (PON-1) activity (patient 1 and kindred, patient 3).

PON1 activity was measured in serum using phenylacetate as substrate [19]. Initial rates of hydrolysis were determined spectrophotometrically at 270 nm. The assay mixture included 1 mM phenylacetate and 0.9 mM CaCl_2_ in 20 mM Tris-HCl, pH 8.0, and 10 µL serum (diluted 1:40). The ɛ_270_ for the reaction was 1310 M^− 1^ cm^− 1^. Arylesterase activity was expressed as the number of micromoles of phenylacetate hydrolyzed per minute per milliliter of serum. To determine the distribution of PON1 in lipoprotein fractions, 300 µL of plasma heparin was separated by size exclusion chromatography using a Bio-Prep SE1000/17 column coupled to a Bio-Rad Duo Flow system as previously described [20] with slight modifications. Briefly, protein elution was accomplished with 2 mM CaCl_2_ in 20 mM Tris-HCl, pH 8.0, at a flow rate of 1mL/min. Fractions of 0.5 mL were collected and PON1 activity was assessed after elution using 10 µL of each fraction. The column was calibrated with VLDL, LDL and HDL isolated by ultracentrifugation from a pool of 5 plasma samples obtained from 5 normolipemic volunteers. For the calibration, cholesterol was determined in the elution fractions by enzymatic colorimetric methods commercially available.

Mutational analysis.

Genomic DNA was extracted from peripheral leucocytes using a Commercial Kit (Qiagen). The DNA was amplified using conventional polymerase chain reaction (PCR) to obtain the corresponding exons, including the exon-intron regions. The products of PCR were amplified using primers as follows: 1 F, CACTCCCACACCAGATAA; 1R TTATGTCGGGGCTTATGC (332 pb) E2-3 F, GGGGAGGGTAAGTGTGCTTT; E2-3R, GTGTGCAGGTACCCTGTGG (600 pb) E4-5 F, TGTGGAGTACCTGGACAGCA; E4-5R, AGGATCAGCTTGGTCTCACC (584 pb) E6F, GAGCCTACACTCAGCAGGTTG; E6R, GTGGCTGGTGAGGAGTGAA (746 pb). This was carried out under the following conditions: 97 °C 7 min per cycle; 95 °C 30 s; 56 °C 30 s; 72 °C 2 min; 40 cycles; 72 °C 10 min per cycle; 4 °C hold. To amplify exon 6, the temperature for alignment was 58 °C. After purification, all DNA fragments were sequenced using forward and reverse primers. The sequencing was performed in an ABI prism 3100 genetic analyzer (Applied Biosystems). The reference sequence was obtained from the National Center for Biotechnology Information (NM_000229.1).

### Statistical analysis

Categorical variables are reported as frequencies and percentages. Continuous data is shown as mean and standard deviation or median and interquartile range depending on the parametric or non- parametric distribution of variables. Categorical variables are compared using the chi-square test or Fisher’s test as appropriate. Students T-test and the U Mann-Whitney test were used for comparisons for parametric and non-parametric variables respectively. A *P*-value < 0.05 was considered as statistically significant. Statistical analyses were performed using Statistical Package for Social Science (IBM Corp. Released 2012. IBM SPSS Statistics for Windows, Version 21.0. Armonk, NY: IBM Corp.) and GraphPad Prism (GraphPad Prism version 7.0.0 for Windows, GraphPad Software, San Diego, California USA).

## Results of the systematic review

**Fig. 1:**
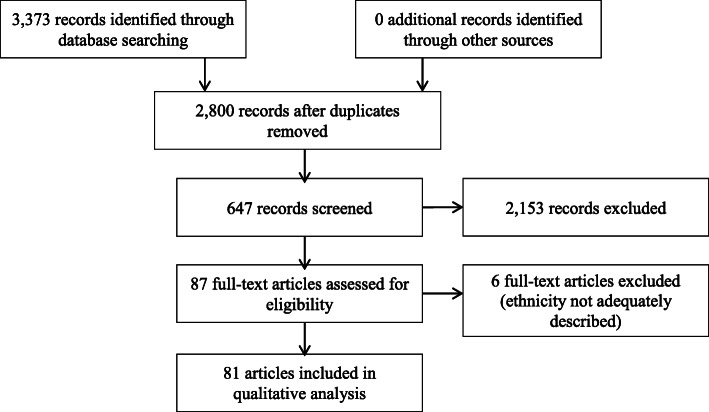
Systematic review

The PRISMA algorithm is shown in Fig. 1. The research strategy retrieved a total of 3,373 publications. After removing any duplicate documents, 2,800 abstracts were reviewed. Of these, 2,153 articles were excluded, as they did not complete inclusion criteria. In total, 87 relevant articles/abstracts were reviewed in detail for eligibility. Of these, six publications were excluded due to incomplete information. Finally, 81 studies were included for the purposes of this article (Table 1, [6, 7, 21–100]).

**Table 1:** Systematic analysis of cases with LCAT deficiency

Ethnicity/ Country	REF	Study Type	Sample Size	Median Age	Gender	Phenotype	HDL-C (mg/dL)	Hemolytic Anemia	Proteinuria/ Albuminuria	eGFR <60 mL/min	LCAT Activity nmol/mL/h	Premature CHD	EXON	Nucleotide change
**Asian**														
Japanese	19–32	CR: 13 R: 1	21	44	F:7 M:11 Unk: 3	FLD:18 FED:3	8.1	No:2 Yes:15 Unk: 4	No:3 Yes:13 Unk: 5	No:12 Yes:4 Unk: 5	7.91	No:16 Unk:5	EX1:3 EX2:4 EX3:1 EX4:3 EX5:1EX6:6	c.367 C > T c.490 C > T c.821 C > G c.101dupC c.491_493dup´GCG c.756 C > A c.951G > A g.501 C > T c.278 C > T T13M 1102G > A c.862delG c.221G > A c.86 A > T Insertion of G-g-C coding glycine at codon 141 c.607G > C c.160G > A c.951G > A
Korean	33	CR: 1	1	33	M:1	FLD:1	12	Yes:1	Yes:1	No:1	0.10	No:1	EX5:1 EX6:1	c.794_801de c.633dupG
**African**														
Morrocan	34	CR: 1	3	17,12,3	F:2 M:1	FLD:3	6	Yes:1 No: 2	Yes:3	No:3	0.39	No:3	EX6:1	c.1010G > A
**Caucasian**														
Australian	35	CR: 1	1	63	F:1	FED:2	4	No:1	No:1	No:1	0.80	No:1	EX4:2	c.503 A > G c.440 C > T
Austrian	36–37	CR: 2	4	42	M:2 Unk:2	FLD:3 U: 1	11.75	No:1 Yes:3	Yes:4	Normal:1 Yes:1 Unk:2	0.08	No:4	EX2:1 EX4:2	c.491G > A c.283G > C
British	38–40	CR: 3	3	58	F:1 M:2	FLD:2 FED:1	3	No:2 Yes:1	No:2 Yes:1	Normal:3	0.47	Present:2 (FLD, FED) No:1	EX1:2 EX4:1	c.54ins30bp c.169G > C c.440 C > T
Canadian	41–42	CR: 2	7	38	F:2 M:5	FLD:3 FED:4	6.14	No:4 Yes:3	No:4 Yes:3	No:5 Yes:2	4.30	No:7	EX4:1 EX6:3	c.1244 A > G c.827T > A c.485 C > T c.1198insG
Caucasian	43–44	CR: 2	4	34	M: 4	FLD: 4	15	Yes:2 Unk:2	No:1 Yes:1 Unk:2	No:2 Unk:2	7.55	No:2 Unk:2	Ex1:2 EX5:1 EX6:3	c.991 C > T c.1216T > G c.703G > A c.1034 C > T c.154G > A c.110 C > T
Danish	41	CR:1	1	47	M:1	FLD:1	7	Yes:1	Yes:1	Yes:1	1.30	No:1	EX3:1 EX5:1	c.544 C > T c.349G > A
Dutch	45–49	CR: 3 R: 1	15	55	F:7 M:8	FED:10 U:5	12	No:10 Unk:5	No:10 Unk:5	No:10 Unk:5	41.71	Present 3 (FED) No:8 Unk:5	EX1:1 EX2:1 EX3:2 EX4:4 EX5:2 EX6:5 IVS4:1	c.403T > A g.2327T > C c.1244 A > G c.101 C > A c.1039 C > T c.476G > A c.736G > T c.802 C > T c.964 C > T c.402G > T c.1012 C > T c.463 A > G c.440 C > T c.296G > C
Finnish	50–52	CR: 2	4	44	F:1 M:3	FLD:4	8	Yes: 4	Yes: 2 No:2	No:4		No:4	EX1:1 EX6:2	c.760G > C c.101insC c.1267 C > T
French	53–57 41	CR: 3	9	43	F:6 M:3	FLD:5 FED:2	5.91	No:4 Yes:4 Unk:1	No:5 Yes:2 Unk:2	No:6 Yes:2 Unk:1	5.57	No:8 Unk:1	EX4:2 EX5:4 EX6:1	c.585T > G c.488_489delTG c.544 C > T c.698T > C c.970-972del c.511 C > T
French Canadian	58	CR: 1	2	25	M: 2	FLD: 2	6.5	Yes:2	Yes:1 No:1	No:2	4.00	No:2	EX1:1	c.102delG
Germany	59–62	CR: 4	5	55	F: 1 M:3	FLD:2 FED: 3	4.33	Yes:2 No:3	Yes:2 No:3	Yes:2 No:3	0.51	Present: 2 (FED) No: 2 Unk:1	EX2:1 EX4:3 EX5:1 EX6:1	c.1112 C > T c.604dupA c.440 C > T c.160G > A
Greek	63	CR: 1	9	49	F:1 M:8	FLD:9	13	Yes:9	Yes:9	Yes:9		No:7	EX6:1	c.802 C > T
Italy	64–71, 41	CR: 6 R: 1	27	33	F: 2 M: 20 Unk:5	FLD: 18 FED:4 U:5	10	No: 4 Yes:16 Unk:7	No:7 Yes:13 Unk:7	No:7 Yes:11 Unk:9	8.96	No:19 Unk:8	Ex1:4 Ex2:2 Ex3:5 Ex4:3 Ex5:2	c.614G > A c.892 A > G c.230 A > G c.1007 A > G c.1021G > A c.1115-1117del c.1132G > A c.1234G > A c.31delG c35C > T c.209T > A c.803G > A c.893 C > T c.997G > A g.131–157 c.343T > C c.1187T > G c.511 C > T c.141-145del c.321 C > A c.1034 C > T c.892 A > G c.321 C > A c.301G > A c.1289 C > T c.726G > C
Norwegian	72,73	CR: 2	9	33	F:6 M:3	FLD:9	11	Yes:9	Yes:8 No:1	Yes:3 No:6	0.4, 1.3	No:6 Unk:3	EX6:4	c.827 T > A c.803 G > A
Polish	74	CR: 1	2	35	F:1 M:1	FLD:2	19	Yes:2	Yes:2	Yes:1 No:1		No:2	EX6:1	c.997G > A
Portuguese	75	CR: 1	2	39	M:2	FLD:2	15	Yes:2	Yes:2	Yes:2		No:2	EX6:1	c.803G > T
Romanian	76	CR:1	1	33	F:1	FLD:1	12	Yes:1	Yes:1	No:1		No:1	EX3:1	c.356G > A
Spanish	77–80	CR: 3 R: 1	12	51	F:3 M:2 Unk:7	FLD:3 FED:1 U:8	10.4	No:2 Unk:10	No:3 Yes:2 Unk:7	No:3 Yes:2 Unk:7	19.36	No:5 Unk:7	EX2:1 EX3:1 EX4:1 EX5:2 IVS3:1	g.2147 C > A c.367 C > T c.285-286del c.512G > A
Swedish	81	CR: 1	3	68	F:3	FED:3	7.3	No:3	No:3	No:3	2.40	Present:1 (FED) No:2	EX1:1	c.101 C > T
**Latin-American**														
Argentinian	82	CR: 1	1	63	F:1	FED:1	4	No:1	No:1	No:1	2.40	Present:1 (FED)		
Brazilian.	83,84	R: 1 A:1	38	38	F: 18 M: 20	FLD: 38	10	Yes: 25 No: 13	Unk:38	Yes: 7 Unk:31		Unk: 38	EX: 5 EX: 6	c.803 G > A c.893 C > T c.679 A > T
Chilean	85	CR: 1	1	36	F:1	FLD:1	3	Yes:1	No:1	No:1		No:1	EX6:2	c.1210 A > G c.997G > A
Colombian	86	CR: 1	1	33	M:1	FLD:1	4	Yes:1	Yes:1	Yes:1		No:1		
Ecuadorian	87	CR: 1	1	60	F:1	FED:1	Unk:1	No:1	No:1	No:1		Present:1 (FED)		
Mexican Mestizo	88	CR: 1	6	42	F:3 M:3	FLD:5 FED:1	8	No:1 Yes:5	No:1 Yes:5	Normal:1 Yes:5	3.33	Present:1 (FED) No:5	EX1:4 EX4:1	c.110 C > T c.490 C > T c.101dupC c.110 C > T Trp8*
**Middle East/south asian**														
Indian	89–91	CR: 3	6	38	F:3 M:3	FLD:4 FED:2	13	Yes:4 No:2	Yes:4 No:2	No:2 Yes:4	28.00	No:6		
Iranian	92	CR: 1	8	28	F:5 M:3	FLD:8	28	No:8	No:8	Yes:8		No:8	EX2:1	c.301G > A
Lebanese	93	CR & R: 1	1	50	M:1	FLD:1	7	Yes:1	Yes:1	Yes:1		No:1	EX6:1	c.950T > G
Pakistani	94	CR: 1	1	33	F:1	FLD:1	13	Yes:1	Yes:1	No:1	0.10	No:1	EX2:1 EX6:1	c.802 C > T c.295T > C
Turkish	95	CR: 1	2	54	F:1 M:1	FED:2	12	No:2	No:2	No:2		Present:2 (FED)	EX1:1 EX6:1	c.1052 A.G c.86 A > G
**Mixed**														
American	96–100	CR: 5	6	33	F:2 M:3 Unk:1	FLD:2 FED:3 U:1	5.62	No:3 Yes:2 Unk:1	No:3 Yes:2 Unk:1	No:5 Unk:1	3.08	No:5 Unk:1	EX1:2 EX2:2 EX3:2 EX5:2 EX6:2	c.110 C > T c.538T > A c.254G > A c.382G > A c.1103G > T c.837_838del c.321 C > A c.576delC c.101 C > T

Abbreviations. *REF* reference. *eGFR* estimated glomerular filtration rate (MDRD equation), *CHD* coronary heart disease, *CR* case report, *R* review, *A* abstract, *FLD* familial LCAT deficiency, *FED* Fish-eye disease, *U* unclassified, *Unk* unknown F: female, *M* male, *EX* exon

The systematic analysis retrieved 215 cases, of which 71.6 % (*n* = 154) were FLD, 19.0 % (*n* = 41) were FED and 9.3 % (*n* = 20) were unclassified (Table 2). Most of the information was found in case reports (87.6 %). The LCAT deficiency cases are from 33 countries, the majority of individuals are Caucasians and the commonest presenting feature was corneal opacity. There is a predominance of men (*n* = 116, 53.9 %) and the mean age of individuals is 42 ± 16.5 years. The median concentration of HDL-C is 7 [4–12] mg/dL, the median LCAT activity is 1.65 (0.0-7.1) nmol/mL/hr and median levels of triglycerides are 206 (138–380) mg/dL. A creatinine clearance < 60mL/min was found in 30.2 %, > 60 in 40.4 % and unknown in the remaining cases. Albuminuria/ proteinuria was present in 39.1 % and absent in 29.8 % of cases. Anemia was reported in 53.9 % and absent in 32.1 %. Premature coronary artery disease was present in 7.4 %, absent in 59.1 % and not evaluated or unknown in the remaining cases.

**Table 2:** Systematic analysis: Characteristics of FLD and FED cases

		Total *n* = 216 (Unclassified *n* = 20)	FLD (*n* = 153, 70.8 %)	FED (*n* = 43, 19.9 %))	p
**AGE (years)**^a^		44.8 (±15.6)	40.2 (±13.8)	53.0 (±15.6)	**<0.001**
**GENDER (*n* = 203)** **(Unknown = 13)**	**MALE**	119 (58.6 %)	93 (60.7 %)	18 (41.8 %)	0.17
	**FEMALE**	84 (41.3 %)	57 (37.2 %)	23 (53.4 %)	
**PREMATURE** **CHD (*n* = 148)** **(Unknown = 68)**	**YES**	13 (6.01 %)	1 (0.65 %)	12 (27.9 %)	**<0.001**
	**NO**	135 (62.5 %)	112 (73.2 %)	16 (37.2 %)	
**HDL-C mg/dL**^b^		7 (4–12)	7.0 (4–12)	6.7 (3.9–9.7)	0.272
**Triglycerides mg/dL**^b^		206 (138–380)	185 (121–380)	283 (164–528)	0.165
**LCAT ACTIVITY nmol/mL/h**^b^ **(*n* = 78)**		1.50 (0.00-7.02)	0.1 (0.0–2.1)	2.1 (0.9–3.5)	**0.013**
**eGFR < 60 (*n* = 184)** **(Unknown = 32)**	**YES**	65 (35.3 %)	62 (40.5 %)	3 (6.9 %)	**0.00**
	**NO**	87 (47.2 %)	48 (31.3 %)	38 (88.3 %)	
**PROTEINURIA/** **MICROALBUMINURIA** **(*n* = 177)** **(Unknown = 29)**	**YES**	84 (47.4 %)	83 (54.2 %)	1 (2.32 %)	**0.00**
	**NO**	64 (36.1 %)	24 (15.6 %)	40 (93.02 %)	
**HEMOLYTIC ANEMIA** **(*n* = 215)** **(Unknown = 30)**	**YES**	116 (53.9 %)	115 (75.1 %)	1 (2.32 %)	**0.00**
	**NO**	69 (32.1 %)	28 (18.3 %)	20 (46.5 %)	

Abbreviations. *FLD* Familial *LCAT* deficiency. *FED* Fish eye disease. *CHD* coronary heart disease. *eGFR* estimated glomerular filtration rate (MDRD equation)

^a^ media (±DE) ^b^ mediana (IIC)

On comparing the individuals with FLD and FED, certain differences are apparent. The FLD cases are significantly younger than the FED cases (41 ± 14.7 vs. 55 ± 13.8 years, *P* = 0.02, respectively). There was no difference in HDL-C levels between groups. However, LCAT activity was significantly lower in FLD compared to FED (0.1 (0.0–2.1) nmol/mL/hr vs. 2.7 (0.8–7.0), *P* = 0.01). Unsurprisingly, clinical features compatible with FLD are significantly more common in these cases (low creatinine clearance, albuminuria/proteinuria and anemia). Premature coronary artery disease was significantly more prevalent in FED compared with FLD (*P* = 0.00). A comparison between the 3 phenotypes available (unclassified, FLD, and FED) is presented in supplementary Table 4. The main characteristics of unclassified patients is also available in supplementary Table 5.

Mutational analysis:

A total of 138 mutations in the *LCAT* gene were recovered (136 in exons and 2 in introns) (supplementary Table 1). Mutations have principally been published in Caucasians. Genetic alterations are present on all exons of the gene; there was no association between a particular exon and phenotype. No specific mutation was associated with an ethnic group. The number of mutations associated with FLD, FED and unclassified cases were 77, 38 and 23 respectively. The FLD phenotype was associated with exon 6 (n = 27), exon 5 (n = 13) and exon 1 (n = 13). In FED, exon 6 (n = 12), exon 4 (n = 10) and exon 1 (n = 8) appeared to have the greatest number of alterations. In unclassified cases, exon 6 was also the predominant site on the *LCAT* gene.

The ethnic distribution of the cases was reviewed with respect to location of LCAT mutation (supplementary Table 1). Here exon 6 (*n* = 41) and exon 1 (*n* = 19) were the most common sites for LCAT mutations. There was a predominance of exon 6 mutations, in Italians, Dutch and Japanese groups. In the Amerindian ethnic group, exon 1 was most common in Mexican-Mestizos whilst exon 6 predominated in Brazil and Chile.

Finally, the number of mutations per exon, adjusted for size of exon was examined (supplementary Table 2). This avoids exon size bias; exon 6 is more than double the length of the others, perhaps explaining the greater number of mutations encountered. With this analysis, a fairer comparison between exons is possible. Exon 4 and exon 1 show the greatest density of mutations, with exons 5 and 6 showing the least number of alterations. There was no clear relationship between the alterations and the key positions for the enzymatic activity of the LCAT protein.

Characteristics of the Latin American cases:

In total, 48 cases of LCAT deficiency have been published from six Latin American countries (Argentina, Brazil, Chile, Columbia, Ecuador and Mexico) (Table 3). There are 38 FLD cases from Brazil (published in an abstract), one unclassified case from Argentina and 3 FLD cases from Chile, Colombia and Ecuador respectively. In Mexico, six cases (4 probands) have been encountered (4 FLD, 1 FED and 1 unclassified); one of which has previously been published (unclassified probable FED).

**Table 3:** Characteristics of Latin America population

Country	Exon mutation	Nucleotide change	Phenotype	Age	Gender	HDL-C mg/dL	TG mg/dL	LCAT activity nmol/mL/h
Argentinean	Unknown	Unknown	Unclassified	63	F	4	765	2.4
Brazilian	Ex 5 Ex 6	c.679 A > T c.803 G > A c.893 C > T	38 FLD	38	18 F, 20 M	<10		-
Chilean	Ex 6 Ex 6	c.997G > A c.1210 A > G	FLD	36	F	3	387	-
Colombian	-	-	FLD	33	M	4	1260	-
Ecuadorian	-	-	FLD	60	F			-
Mexican Mestizo (1 proband and 2 other members of same family)	Ex 1	Trp8*	FLD	37	F	15	300	3.70
Mexican Mestizo	Ex 1 Ex 1	c.101dupC c.110 C > T	FED	70	F	12	334	4.20
Mexican Mestizo	Ex 1	c.110 C > T	FLD	34	M	2	597	2.10
Mexican Mestizo	Ex 4	c.490 C > T	FLD	29	F	4	186	-
**Total**		**10**	**48**	**-**	**-**	**-**		**-**

Abbreviations. *HDL-C* high density lipoprotein cholesterol, *TG* triglycerides

The mean age of the cases was 45 years (in Brazil it was 38 years) with an equal sex distribution. The mean HDL-C level was 5.4 (in Brazil it was < 10 mg/dL) and LCAT activity was reported in only 4 individuals with a mean level of 3.1nmol/mL/hr.

Molecular analysis of the Mexican- Mestizo patients revealed mutations in exon 1 (Trp8*, c.101dupC, c.110 C > T) in 3 probands, and a homozygous alteration on exon 4 in 1 proband (c.490 C > T). The Chilean case reported alterations on exon 6, c.1210 A > G and c.997G > A. In Brazil, 38 cases have been reported; the investigators encountered three pathogenic mutations in the LCAT gene, each corresponding to a distinct geographic disease cluster. Two mutations are on exon 6 (c.803 G > A and c.893 C > T) and one on exon 5 (c.679 A > T). Finally, molecular analysis was not carried out in Argentina, Colombia and Ecuador.

### Mexican probands with lcat deficiency syndromes

#### 1. PROBAND 1: Familial LCAT deficiency (FLD)

The proband was a 37-year old woman with bilateral corneal opacities (no deficit in visual acuity). She came from a small village in the state of Oaxaca, in south-west Mexico. She was the 6th of 10 children and her parents were apparently non-consanguineous. Only her paternal grandmother had eyes similar to hers. Of her 9 siblings, 2 brothers had corneal opacities and nephrotic syndrome. There was no history of cardiovascular disease in her family. All available family members, including her parents and 5 of their 10 children were evaluated.

She had a history of hyperlipidemia, arterial hypertension and nephrotic syndrome; a renal biopsy reported glomerulopathy characterized by mesangial proliferation, vacuolated macrophages and presence of intramembranous lipid deposits in glomerular capillaries. A recent carotid doppler ultrasound was normal with no alteration in carotid-intima thickness.

Biochemical analysis:

Laboratory results showed a normochromic, normocytic anemia [hemoglobin 9.2 g/dL (normal 13-15 g/dL)] and measurement of erythrocyte osmotic fragility confirmed the presence of brittle cells. There was evidence of renal failure with nephrotic syndrome (creatinine clearance 47mL/min, and proteinuria of 5 g/24hrs). The lipid profile showed low HDL-C, hypertriglyceridemia and low levels of apolipoprotein A1 (Table 4). The LCAT activity was low (LCAT activity 0.4 %, specific activity 3.7 nmol/mL/hr) and there was a reduction in paroxonase-1 activity (27.8 mU/ml/h, control = 100.77 mU/ml/h).

**Table 4:** Biochemical results of probands 1, 2 and 3 and their kindred

		TG	Total chol	HDL-C	LDL-C	Apo A1	Apo B	LCAT activity %	Specific LCAT Activity (nmol/mL/h)	PON-1 mU/ml/h (Control 100.77)
**PROBAND 1**	Father (II-1)	112	168	37	109	132	92.5	2.70	25.63	100.5
	Mother (II-2)	188	203	38	127	135	122	3.18	30.19	62.6
	**Proband (**37 yrs) (III-7)	**300**	**153**	**15**	**78**	**44**	**113**	**0.4**	**3.79**	**27.8**
	Brother1 (35 yrs) (III-2)	1076	222	**11**	---	**35.3**	52.1	**0.9**	**8.54**	**42.6**
	Brother2 (33 yrs) (III-1)	589	313	**17**	178	**47.2**	114	**0.5**	**4.74**	**53.0**
	Brother3 (22 yrs) (III-10)	117	161	26	112	91.6	102	2.42	22.98	97.0
	Sister (45 yrs) (III-5)	177	168	23	110	---	---	3.82	36.27	81.7
**PROBAND 2**	**Proband** (II-1)	**334**	**150**	**12**	**71**	**48.7**	**109**		**4.2**	
	Daughter 1 (III-1)	290	196	30	108	131	116		55	
	Daughter 2 (III-2)	191	157	25	94	123	108		52.7	
	Granddaughter (mother is daughter 1) (IV-1)	59	180	81	87	210	52.7			
	Grandson (mother is daughter 1) (IV-2)	450	240	48	102	169	121			
	Grandson (mother is daughter 2) (IV-4)	85	162	46	99	157	92.3			
**PROBAND 3**	**Proband (II-1)**	**186**	**117**	**4**	**59**				7.3	
	Father (II-1)	257	168							

Abbreviations. TG: triglycerides HDL-C: high density lipoprotein cholesterol. LDL-C: low density lipoprotein cholesterol. ApoA1: apolipoproten A1 ApoB: apolipoprotein B

Two brothers were affected (homozygotes) and heterozygote family members had half- normal HDL-C concentrations (Table 4). All three affected individuals showed some paroxonase-1 activity, whereas LCAT activity was virtually absent. The proband had a 72 % reduction in PON-1 activity, while her affected brothers showed a 47 and 57 % reduction respectively. The 2 remaining siblings and both parents had low LCAT activity (2.4–3.8 %) and higher paroxonase activity compared to the affected individuals. On separation of the lipoproteins by exclusion chromatography, the paroxonase-1 activity was essentially on HDL, with little activity on LDL.

Mutational analysis:

A novel mutation was encountered in this proband. This was a nucleotide replacement resulting in a stop codon at position 8 on exon 1 of the *LCAT* gene (in the leader sequence). Tryptophan (TGG) was replaced by Ambar stop (TAG). This is reported as Trp8* or Trp-17* (*indicates stop codon) in the nucleotide sequence. The parents were heterozygous for the mutation and the proband and both her affected brothers were homozygous. The family pedigree is shown in supplementary Fig. 1.

#### 2. PROBAND 2: Fish Eye Disease (FED)

The 70-year-old proband from Mexico City, had bilateral corneal opacities and a history of myocardial infarction. The subject’s father and mother had suffered from coronary artery disease (her father died at age 66, her mother died at age 70). The only other family members with similar eyes were her father, paternal grandmother and one male sibling who died soon after birth. None of her 8 siblings were alive. Both her children and 5 grandchildren had normal corneas and no health issues. The proband, her two daughters and 4 of her grandchildren were studied.

The proband had type 2 diabetes mellitus (no known complications), mixed hyperlipidemia, and arterial hypertension (history of atrial fibrillation and left ventricular hypertrophy). The coronary heart disease was characterized by occlusion of 3 coronary vessels (left coronary: trunk, circumflex and right coronary); she had been treated with two medicated stents.

Biochemical analysis:

The lipid profile showed an HDL-C level of 11 mg/dL (Table 4). The remaining laboratory results included; glucose 102 mg/dL, creatinine 0.87 mg/dL, hemoglobin 12.1 g/dL. The proband had no evidence of anemia or renal disease.

No other family member had a clinical or biochemical phenotype compatible with FED. The proband and her daughters had HDL cholesterol levels < 40 mg/dL, while all the grandchildren had normal HDL-C levels. The proband had low levels of apolipoprotein A1; her daughters had levels intermediate between hers and those of her grandchildren. The proband had extremely low LCAT activity (LCAT specific activity 4.2 nmol/mL/hr) and both daughters had relatively normal LCAT activity (55.0 and 52.7 nmol/mL/hr respectively, Control = 78.7).

Mutational analysis:

Two mutations were found on exon 1:

1. On one allele, there was an insertion of cysteine (reported as c.101dupC) at codon 34. This mutation resulted in a stop codon 7 codons later. The proband was heterozygote for this mutation.
2. On the other allele, the alteration was c.110 C > T. When this allele is translated, threonine is substituted by methionine (ACG-ATG Thr37Met) (missense mutation) at position 37 of the protein. The proband was heterozygote for this mutation.

The proband is compound heterozygote for both mutations. Daughter 1 is heterozygote for the second mutation (c.110 C > T at codon 37), while daughter 2 is heterozygote for the first mutation (c.101dupC at codon 34). Analysis of the apoA1 gene and its promotor region was also carried out in the proband, no alterations were found.

The family pedigree is shown in supplementary Fig. 1.

#### 3. PROBAND 3 (Familial LCAT Deficiency)

The proband was a 29-year-old woman from Monterrey, with bilateral corneal opacities resembling premature corneal arcus. She had an FLD phenotype with extremely low levels of HDL-C, anemia and kidney disease (glomerulopathy).There was no clinical or biochemical evidence of FLD or FED in her parents or sibling. There was no family history of premature cardiovascular disease. She had attended consultations with several specialists, had undergone three kidney biopsies and one bone marrow aspiration; despite this she had not been diagnosed.

Biochemical analysis.

The proband had an HDL-C level of 4 mg/dL (Table 4). The laboratory profile showed: glucose 101 mg/dL, creatinine 1.12 mg/dL, hemoglobin 12.9 g/dL and 24-hour urinary protein 2307 mg/day. The proband had extremely low LCAT activity (7.3 nmol/mL/hr, LCAT specific activity in control 145.34nmol/mL/hr) and a 60 % reduction in PON-1 activity compared with controls.

Mutational analysis:

The genetic alteration was a point mutation in exon 4 of the LCAT gene, i.e., a G to A substitution on codon 140 converting Arginine to Histidine. The family pedigree is shown in supplementary Fig. 1.

## Discussion

FLD and FED are rare LCAT deficiency syndromes with differing clinical manifestations. This is the first systematic analysis evaluating the ethnic distribution of LCAT deficiency syndromes, with particular emphasis on Latin America, presenting the case histories of three Mexican-Mestizo probands.

The systematic review retrieved 215 published cases of which 71.6 % were reported as FLD, 19 % as FED and 9.4 % were unclassified. This number is significantly greater than that reported in the current literature [101]. The majority of probands have been published in case reports, often with incomplete clinical or genetic information. This disease continues to be diagnosed late, with corneal opacifications being the principal reason for consultation. FED is diagnosed significantly later than FLD, probably because the severe clinical phenotype associated with the later results in earlier medical attention.

LCAT enzyme activity was significantly lower in FLD compared to FED however there was no difference in HDL-C levels between the two phenotypes. Indeed, both can have similar lipid profiles, suggesting any variability in parameters is unrelated to LCAT function. Pavanello et al. commented that the severity of the hypoalphalipoproteinemia varies widely among carriers of different LCAT genotypes [101]. Furthermore, carriers of one mutant LCAT allele show an intermediate biochemical phenotype between homozygous carriers and controls, suggesting that the disease, which is reported as recessive, is indeed co-dominant for the biochemical phenotype.

The clinical features of the probands showed a clear difference between FED and FLD, with renal disease and anemia prevalent in the later. Renal damage is the principle cause of morbidity and mortality in FLD. This usually begins as proteinuria in childhood and progresses to renal failure in the fourth decade. The deposition and accumulation of nephrotoxic and pro-inflammatory lipoprotein X (Lp-X) particles, mainly in the mesangium, in the absence of LCAT, may explain the development of renal disease [14]. However, not all cases of FLD showed significant proteinuria or reduced eGFR; this suggests that the rate of progression to renal failure may be highly variable. Lamiquiz-Moneo et al. state that this clinical variability is likely to be related to the biochemical /lipid phenotype rather than to the inherited mutation [80]. In addition, an unclassified clinical phenotype was present in 9.3 % of our cases; the authors could not confirm either FLD or FED (supplementary Tables 4 and 5). Some authors have commented that the clinical manifestations of patients with LCAT gene mutations may vary even among members of the same family carrying identical mutations [43]. Mahapatra et al., have reported the co-existence of differing phenotypes in the same family; they report a proband with FLD, while the sister and mother presented with FED. [88]. Thus, LCAT deficiency syndromes appear to show both biochemical and clinical heterogeneity.

There was a significantly greater prevalence of premature CHD in FED patients compared to FLD patients. The cardiovascular risk associated with LCAT deficiency syndromes is still a matter of debate. [83]. Oldoni et al., compared carotid intima media thickness between 33 heterozygous FLD subjects and 41 heterozygous FED subjects [102]. Carriers of FLD mutations exhibited less carotid atherosclerosis, whereas those with FED mutations presented with more subclinical atherosclerosis. This may be related to the capacity of LCAT to esterify cholesterol on apolipoprotein B–containing lipoproteins- this is lost in FLD, but is unaffected in FED. In a study of Italian FLD families, the inheritance of a mutated LCAT genotype had a gene-dose dependent effect in reducing carotid IMT, however, a subgroup of these carriers also showed normal flow-mediated dilation [65, 83]. In general there are few longitudinal follow-up studies hence a definitive conclusion is hard to reach.

The molecular defects associated with LCAT deficiency syndromes show heterogeneity. In total, 138 LCAT mutations were found with no particular exon dominating in a particular ethnicity. There was no association between clinical phenotype and genetic alteration, this may be due to the low number of cases worldwide. Exon 6 was the predominant site for both FLD and FED; however, after adjusting for exon size, exon 1 and 4 showed the greatest concentration of mutations. At present, it is impossible to predict the phenotype (FLD or FED) associated with LCAT mutation [101].

With regards to ethnicity, at least 33 different groups are represented, of which Caucasians are most common. The predominance of Caucasian and Asian cases may reflect better health awareness and access to health care in these regions. Only one case has been reported in the African sub-continent. Cases are more likely in countries hosting research groups with interest and resources to investigate this disease. At present, only six countries have reported probands with LCAT deficiency in Latin America, and genetic evaluation has only been carried out in three [82–88]. Of these, Brazil reports three mutations causing FLD each associated with a distinct geographical region. In Mexico, the first FLD proband, came from an isolated village in the south of Mexico, there is little genetic admixture in this region. The indigenous heritage of this patient may have been responsible for disease susceptibility. The mutations reported in Brazil and Chile are on exon 5 and 6. In Mexico, exon 1 mutations predominate (3 probands), with only 1 case from the north of the country showing a homozygous exon 4 alteration.

The first Mexican- Mestizo proband (FLD phenotype) showed an alteration located in the leader sequence of the gene, thus normal protein synthesis is abolished. To our knowledge, this particular mutation is novel, only one other mutation in this region of the gene has been reported: Calabresi et al. mention a subject with a Thr-13Met mutation and an FLD phenotype [64]. The proband continued to show paroxonase- 1 activity, essentially on HDL, even though the number of particles was extremely low and despite a clear lack of LCAT activity. The proband had 27 % activity, while her affected siblings had approximately 50 % activity. The heterozygote family members had essentially normal PON-1 activity. This enzyme prevents the conversion of LDL into a more atherogenic particle [103]. Preserved PON-1 activity has been reported in other HDL-C deficiency states, and in vitro experiments with LCAT deficient plasma suggest an apparent maintenance of cholesterol efflux [94, 104, 105]. Reverse cholesterol transport and protection of LDL from oxidative stress is possibly conserved in complete LCAT deficiency, supporting the differential cardiovascular risk between phenotypes.

The second Mexican- Mestizo proband (FED) had two distinct *LCAT* mutations, one on each allele (compound heterozygote). One of the alterations was a frameshift mutation (c.101dupC) on exon 1; the other mutation was a missense mutation (c.110 C > T) on the same exon. Both mutations have previously been reported in the literature [27–29].

Argyropoulos et al. have reported an FLD Caucasian proband who was compound heterozygote with a missense mutation identical to that in the proband (c.110 C > T on exon 1) [46]. Posadas-Sanchez et al. have reported the presence of the same missense mutation (c.110 C > T) on both alleles (homozygote) in an unrelated Mexican subject with an unclassified LCAT deficiency syndrome (probable FED) [88]. The 34- year old Mexican man had type 2 diabetes, premature coronary artery disease, corneal opacities, normal renal function and extremely low levels of HDL cholesterol (2 mg/dL). The investigators reported an increased number of small HDL particles, which had a reduced ability to promote cholesterol efflux (PON-1 activity was low). Finally, Bujo et al. have published the presence of the homozygous c.101dupC mutation on exon 1 in a Japanese subject. This resulted in a truncated 16 amino-acid non -functional LCAT protein and an FLD phenotype [22]. Predicting the effect of the co-existence of two different mutations (one on each allele) on LCAT function and structure is not straightforward. The majority of mutations are not located in sites involved in the catalytic function of the enzyme; the affected sites are probably involved in maintaining protein stability and structure. The mature LCAT protein contains 416 amino acids and a leader sequence (67 kDa) [106–108]. LCAT has two disulfide bridges between Cyst50-Cys74 and Cys313-Cys356; the first bridge partially covers the active site of LCAT, forms part of the lid region and is thought to enable the enzyme to bind to lipid surfaces. In our patient, the genetic alterations may interfere with the nearby lid structure or produce a conformational change when the mature protein is folded, resulting in enzyme- substrate interference. The frameshift mutation is a more detrimental alteration; however, clinical expression of which would only be apparent in homozygotes. Hence, the predominant phenotype in the subject is FED.

The third Mexican-Mestizo proband (FLD), had a point mutation on exon 4 of the *LCAT* gene; this mutation has been reported in an Austrian kindred who were homozygous for this modification [39]. This domain (where Arg140 resides) is crucial for an enzymatically active LCAT protein, mutations in this region possibly affect tertiary structure.

In Latin America, persons with LCAT deficiency syndromes face unique challenges. The medical community is unaware of this condition; the third proband had attended consultations with several specialists and had not been diagnosed opportunely. Many centers do not have the infrastructure for the biochemical or genetic studies necessary to confirm this condition. Current management of FLD is preventative and involves lipid lowering therapy, ACE inhibitors, diuretics and steroids, in order to delay progression to end-stage renal disease: for many in Latin America, these medications will be an out of pocket expense. Furthermore, access to further treatment with peritoneal dialysis or hemodialysis is variable. Provision of renal replacement therapy (RRT) has increased in all Latin American countries over the past 20 years, universal access is available in only a few countries (Argentina, Brazil, Chile, Cuba, Uruguay, Venezuela, and Colombia) [109]. Kidney transplantation may offer a temporary cure, but reoccurrence of nephropathy is inevitable [110]. Currently, trials are underway with human recombinant LCAT enzyme and there is the possibility of gene therapy in the future [111]. However, such products maybe unavailable and/or unaffordable in Latin America [112].

### Study strengths and limitations

 This study is the first systematic review of LCAT deficiency syndromes evaluating the ethnic distribution of this condition. This work highlights the major knowledge gaps in this disease. Limitations include the lack of standardized data in the case reports. This limited the analysis in the systematic review. A quality assessment and critical appraisal of the included case reports was also carried out (using the JBI critical appraisal checklist) by two of the researchers and is available in supplementary Table 8 [113]. It is evident that the included articles focus only on the history and diagnosis with little information on treatment and long-term follow-up. This lack of longitudinal data does not allow the natural history of this disease to be examined adequately. Measurements of free cholesterol and cholesteryl ester, as well as cholesterol esterification rate to complete the biochemical characterization of the Mexican probands and their families would have been desirable.

## Conclusions

In conclusion, the systematic review shows that LCAT deficiency syndromes are diagnosed late; with FLD cases identified significantly earlier than FED. This review confirms that this condition is clinically and genetically heterogeneous. There was no association between ethnicity and LCAT mutations. However, there was a significantly greater risk of premature coronary artery disease in FED compared to FLD. This finding is clinically important, it suggests that management should be tailored according to the LCAT deficiency profile. In FLD patients, the priority is to mitigate both CVD and progression to end stage kidney disease; in contrast, in FED patients, management of cardiovascular risk may well be paramount. Finally, the LCAT mutations discussed in this article are the only ones reported in the Mexican- Amerindian population. The novel mutation associated with FLD in a Mexico-Mestizo woman, may suggest the influence of Amerindian ancestry.

## Supplementary Information

**Additional file 1:**

**Additional file 2:**

## Data Availability

all data generated or analyzed during this study are included in this published article [and its supplementary information files].

## Abbreviations

TC: Total cholesterol
PON-1: Paroxonase-1
FLD: Familial LCAT deficiency
FED: Fish Eye disease
LCAT: Lecithin–cholesterol acyltransferase
HDL: High density lipoproteins
LDL: Low density lipoprotein
VLDL: Very low density lipoprotein
apo A-I: Apolipoprotein A-I
apo A-II: Apolipoprotein A-II
