# Supplementary material for: LCAT deficiency: a systematic review with the clinical and genetic description of Mexican kindred
Source: Lipids Health Dis. 2021 Jul 13;20:70. doi: 10.1186/s12944-021-01498-6 (PMC8276382; doi:10.1186/s12944-021-01498-6)
Supplement: Supplementary file 2 — Additional file 2: [file 12944_2021_1498_MOESM2_ESM.docx]

**Supplementary table 1: PRISMA checklist**

| **Section/topic** | **#** | **Checklist item** | **Reported on page #** |
| --- | --- | --- | --- |
| **TITLE** | | |  |
| Title | 1 | Identify the report as a systematic review, meta-analysis, or both. | 1 |
| **ABSTRACT** | | |  |
| Structured summary | 2 | Provide a structured summary including, as applicable: background; objectives; data sources; study eligibility criteria, participants, and interventions; study appraisal and synthesis methods; results; limitations; conclusions and implications of key findings; systematic review registration number. | 2-3 |
| **INTRODUCTION** | | |  |
| Rationale | 3 | Describe the rationale for the review in the context of what is already known. | 5-6 |
| Objectives | 4 | Provide an explicit statement of questions being addressed with reference to participants, interventions, comparisons, outcomes, and study design (PICOS). | 6 |
| **METHODS** | | |  |
| Protocol and registration | 5 | Indicate if a review protocol exists, if and where it can be accessed (e.g., Web address), and, if available, provide registration information including registration number. Registration Number: CRD42021229254. | 7 |
| Eligibility criteria | 6 | Specify study characteristics (e.g., PICOS, length of follow-up) and report characteristics (e.g., years considered, language, publication status) used as criteria for eligibility, giving rationale. | 6 |
| Information sources | 7 | Describe all information sources (e.g., databases with dates of coverage, contact with study authors to identify additional studies) in the search and date last searched. | 6 |
| Search | 8 | Present full electronic search strategy for at least one database, including any limits used, such that it could be repeated. | 6 |
| Study selection | 9 | State the process for selecting studies (i.e., screening, eligibility, included in systematic review, and, if applicable, included in the meta-analysis). | 6 |
| Data collection process | 10 | Describe method of data extraction from reports (e.g., piloted forms, independently, in duplicate) and any processes for obtaining and confirming data from investigators. | 6-7 |
| Data items | 11 | List and define all variables for which data were sought (e.g., PICOS, funding sources) and any assumptions and simplifications made. | 6-7 |
| Risk of bias in individual studies | 12 | Describe methods used for assessing risk of bias of individual studies (including specification of whether this was done at the study or outcome level), and how this information is to be used in any data synthesis. | 6-7 |
| Summary measures | 13 | State the principal summary measures (e.g., risk ratio, difference in means). | NA |
| Synthesis of results | 14 | Describe the methods of handling data and combining results of studies, if done, including measures of consistency (e.g., I^2^) for each meta-analysis. | NA |

Page 1 of 2

| **Section/topic** | **#** | **Checklist item** | **Reported on page #** |
| --- | --- | --- | --- |
| Risk of bias across studies | 15 | Specify any assessment of risk of bias that may affect the cumulative evidence (e.g., publication bias, selective reporting within studies). |  |
| Additional analyses | 16 | Describe methods of additional analyses (e.g., sensitivity or subgroup analyses, meta-regression), if done, indicating which were pre-specified. | 9 |
| **RESULTS** | | |  |
| Study selection | 17 | Give numbers of studies screened, assessed for eligibility, and included in the review, with reasons for exclusions at each stage, ideally with a flow diagram. | 9 |
| Study characteristics | 18 | For each study, present characteristics for which data were extracted (e.g., study size, PICOS, follow-up period) and provide the citations. | 10 |
| Risk of bias within studies | 19 | Present data on risk of bias of each study and, if available, any outcome level assessment (see item 12). | NA |
| Results of individual studies | 20 | For all outcomes considered (benefits or harms), present, for each study: (a) simple summary data for each intervention group (b) effect estimates and confidence intervals, ideally with a forest plot. | NA |
| Synthesis of results | 21 | Present results of each meta-analysis done, including confidence intervals and measures of consistency. | NA |
| Risk of bias across studies | 22 | Present results of any assessment of risk of bias across studies (see Item 15). | 9-12 |
| Additional analysis | 23 | Give results of additional analyses, if done (e.g., sensitivity or subgroup analyses, meta-regression [see Item 16]). | 9-12 |
| **DISCUSSION** | | |  |
| Summary of evidence | 24 | Summarize the main findings including the strength of evidence for each main outcome; consider their relevance to key groups (e.g., healthcare providers, users, and policy makers). | 17-23 |
| Limitations | 25 | Discuss limitations at study and outcome level (e.g., risk of bias), and at review-level (e.g., incomplete retrieval of identified research, reporting bias). | 23-24 |
| Conclusions | 26 | Provide a general interpretation of the results in the context of other evidence, and implications for future research. | 24 |
| **FUNDING** | | |  |
| Funding | 27 | Describe sources of funding for the systematic review and other support (e.g., supply of data); role of funders for the systematic review. | 25 |

*From:*  Moher D, Liberati A, Tetzlaff J, Altman DG, The PRISMA Group (2009). Preferred Reporting Items for Systematic Reviews and Meta-Analyses: The PRISMA Statement. PLoS Med 6(7): e1000097. doi:10.1371/journal.pmed1000097

For more information, visit: **www.prisma-statement.org**.

Page 2 of 2

**Supplementary table 2:** Distribution of LCAT mutation (exon) by ethnicity/race

|  | Exon number | | | | | |  |  |  |  |
| --- | --- | --- | --- | --- | --- | --- | --- | --- | --- | --- |
|  | **1** | **2** | **3** | **4** | **5** | **6** | **IVS3** | **IVS4** | **NRM** | **Total** |
| Asian | **3** | **4** | **1** | **3** | **2** | **7** | **-** | **-** | 1 | **20** |
| JAPANESE | 3 | 4 | 1 | 3 | 1 | 6 | - | - | 1 | 18 |
| KOREAN | - | - | - | - | 1 | 1 | - | - | - | 2 |
| Black | **-** | - | - | - | - | **1** | **-** | **-** | **-** | **1** |
| MORROCAN | - | - | - | - | - | 1 | - | - | - | 1 |
| Caucasian | **12** | **6** | **10** | **19** | **13** | **32** | **1** | **1** | **-** | **94** |
| AUSTRALIAN | - | - | - | 2 | - | - | - | - | - | 2 |
| AUSTRIAN | - | 1 | - | 2 | - | - | - | - | - | 3 |
| BRITISH | 2 | - | - | 1 | - | - | - | - | - | 3 |
| CANADIAN | - | - | - | 1 | - | 3 | - | - | - | 4 |
| CAUCASIAN | 2 | - | - | - | 1 | 3 | - | - | - | 6 |
| DANISH | - | - | 1 | - | 1 | - | - | - | - | 2 |
| DUTCH | 1 | 1 | 2 | 4 | 2 | 5 | - | 1 | - | 16 |
| FINNISH | 1 | - | - | - | - | 2 | - | - | - | 3 |
| FRENCH | - | - | - | 2 | 4 | 1 | - | - | - | 7 |
| FRENCH CANADIAN | 1 | - | - | - | - | - | - | - | - | 1 |
| GERMANY | - | 1 | - | 3 | 1 | 1 | - | - | - | 6 |
| ITALY | 4 | 2 | 5 | 3 | 2 | 13 | - | - | - | 29 |
| NORWEGIAN | - | - | - | - | - | 1 | - | - | - | 1 |
| GREEK | - | - | - | - | - | 1 | - | - | - | 1 |
| POLISH | - | - | - | - | - | 1 | - | - | - | 1 |
| ROMANIAN | - | - | 1 | - | - | - | - | - | - | 1 |
| SPANISH | - | 1 | 1 | 1 | 2 | - | 1 | - | - | 6 |
| SWEDISH | 1 | - | - | - | - | - | - | - | - | 1 |
| PORTUGUESE | - | - | - | - | - | 1 | - | - | - | 1 |
| Latin-American | **4** | **-** | - | **1** | **-** | **3** | **-** | **-** | 3 | **8** |
| ARGENTINIAN | - | - | - | - | - | - | - | - | 1 | 1 |
| BRAZILIAN | - | - | - | - | - | 1 | - | - | - | 1 |
| COLOMBIAN | - | - | - | - | - | - | - | - | 1 | 1 |
| ECUADORIAN | - | - | - | - | - | - | - | - | 1 | 1 |
| MEXICAN MESTIZO | 4 | - | - | 1 | - | - | - | - | - | 5 |
| CHILEAN | - | - | - | - | - | 2 | - | - | - | 2 |
| Middle East/south asian | **1** | **2** | **-** | - | - | **3** | **-** | **-** | 3 | **6** |
| LEBANESE | - | - | - | - | - | 1 | - | - | - | 1 |
| PAKISTANI | - | 1 | - | - | - | 1 | - | - | - | 2 |
| IRANIAN | - | 1 | - | - | - | - | - | - | - | 1 |
| TURKISH | 1 | - | - | - | - | 1 | - | - | - | 2 |
| INDIAN |  |  |  |  |  |  |  |  | 3 | 0 |
| Mixed | **2** | **2** | **1** | **1** | **1** | **2** | **-** | **-** | **-** | **9** |
| AMERICAN | 2 | 2 | 1 | 1 | 1 | 2 | - | - | - | 9 |
| Total | **22** | **13** | **13** | **23** | **17** | **47** | **1** | **1** | **3** | **138** |

**Supplementary table 3.** The number of mutations per exon adjusted for size of exon

| **Exón** | **# mutations per**  **exon**  **FLD FED** | | **Exon size** | **% mutated exon**  **(#mut/exon size)**  **FLD FED** | | **Key positions**  **(codon number)** |
| --- | --- | --- | --- | --- | --- | --- |
| 1 | 13 | 8 | 173 | 7.4% | 4.6% |  |
| 2 | 7 | 1 | 157 | 4.4% | 0.6% |  |
| 3 | 6 | 4 | 116 | 5.1% | 3.4% | 173 |
| 4 | 11 | 10 | 96 | 11.4% | 10.4% | 205 |
| 5 | 13 | 2 | 225 | 5.7% | 0.8% | - |
| 6 | 27 | 12 | 729 | 3.7% | 1.6% | 369, 401 |

**Supplementary Figure 1**: Pedigree studies of 3 Mexican Probands

Pedigree proband 1 (FLD)


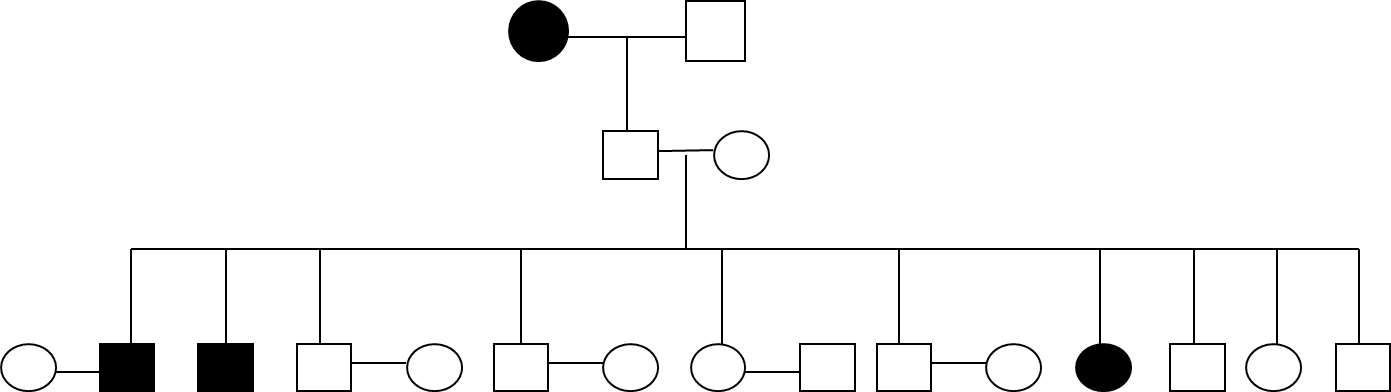


**III-4**

**III-3**

**III-2**

**III-1**

**I-1**

**III-10**

**III-9**

**III-8**

**I-2**

**II-1**

I

II

III

**III-6**

**III-5**

Proband III-7

I

Pedigree proband 2 (FED)

**I-2**

**I-1**

I

**II-2**

**II-3**

**II-1**

II

**III-2**

**III-1**

III

IV

**IV-5**

**IV-4**

**IV-3**

**IV-1**

**IV-2**

Pedigree proband 3 (FLD)


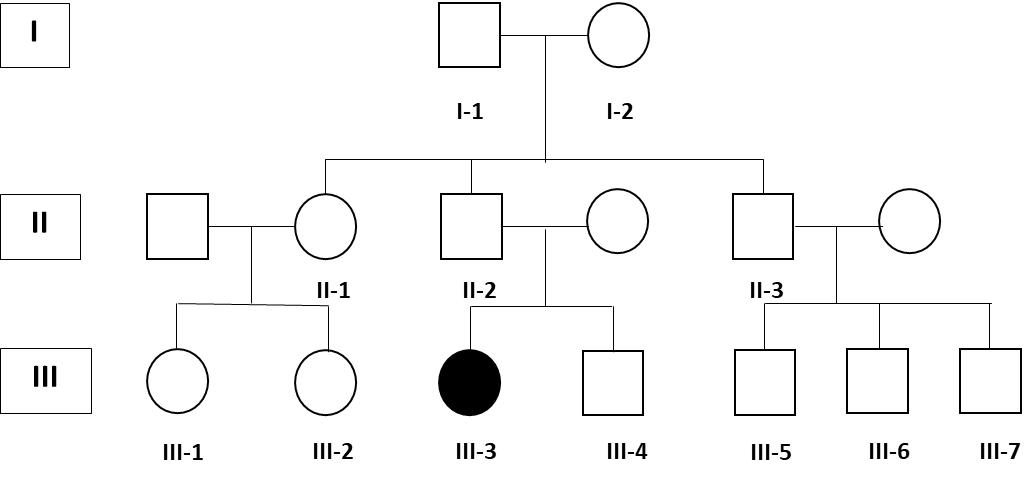


**Supplementary table 4:** comparison of the 3 phenotypes: unclassified, FLD and FED

|  | | **Unclassified**  **(n=20, 9.25%)** | **FLD**  **(n=153, 70.8%)** | **FED**  **(n=43, 19.9%))** | **p** |
| --- | --- | --- | --- | --- | --- |
| **AGE (years)** ^a^ | | 48.5(±16.5) | 40.2 (±13.8) | 53.0 (±15.6) | **<0.001** |
| **GENDER (n=203)**  **(Unknown=13)** | **MALE** | 8  (40%) | 93  (60.7%) | 18  (41.8%) | 0.17 |
|  | **FEMALE** | 4  (20%) | 57  (37.2%) | 23  (53.4%) |  |
| **PREMATURE**  **CHD (n=148)**  **(Unknown=68)** | **YES** | 0 | 1  (0.65%) | 12  (27.9%) | **<0.001** |
|  | **NO** | 7  (35%) | 112  (73.2%) | 16  (37.2%) |  |
| **HDL-C mg/dL** ^b^ | | 19.5  (9.25-24.5) | 7.0  (4-12) | 6.7  (3.9-9.7) | **0.001** |
| **Tryglicerides mg/dL** ^b^ | | 204  (138-267) | 185  (121-380) | 283  (164-528) | 0.225 |
| **LCAT ACTIVITY nmol/mL/h** ^b^  **(n=78)** | | 41.5  (5.5-67) | 0.1  (0.0 – 2.1) | 2.1  (0.9 – 3.5) | **<0.001** |

^a^ media (±DE)

^b^ mediana (IIC)

**Supplementary table 5:** Main characteristics of unclassified patients.

| **Author** | **Country** | **Age** | **Gender** | **CHD** | **HDL-C mg/dL** | **Tryglicerides mg/dL** | **LCAT ACTIVITY nmol/mL/h** | **eGFR <60** | **Anemia** | **Proteinuria** | **Mutation** |
| --- | --- | --- | --- | --- | --- | --- | --- | --- | --- | --- | --- |
| Calabresi | Italy |  |  | Unk |  |  | 16.5 | Unk |  |  | c35C>T |
| Calabresi | Italy | Unk | Unk | Unk | Unk | Unk | Unk | Unk | Unk | Unk | c.230A>G |
| Cohen | USA |  |  |  |  |  |  |  |  |  | c.254G>A |
| Horl | Austria | 32 | M | Unk | 15 | 193 | Unk | N | N | Unk | c.283G>C, c.491G>A |
| Cirera | Spain | 7 | F | Unk | 8 | 141 | 4.5 | N | N | N | c.285-286del |
| Holleboom | Holland | 34 | M | Unk | 4 | 205 | 7.1 | N | N | N | c.296G>C |
| Cohen | USA | Unk | Unk | Unk | Unk | Unk | Unk | Unk | Unk | Unk | c.382G>A |
| Holleboom | Holland | 52 | M | Unk | 23 | 204 | 71 |  |  |  | c.402G>T, c.403T>A |
| Recalde | Spain | 43 | M | Unk | 13 | 282 | 37 | Unk | Unk | Unk | c.512G>A |
| Recalde | Spain | 60 | M | Unk | 28 | 138 | 70 | Unk | Unk | Unk | g.2147C>A |
| Holleboom | Holland | 66 | M | Unk | 22 | 206 | Unk | Unk | Unk | Unk | c.736G>T |
| Holleboom | Holland | 52 | M | Unk | 17 | 312 | 46 | Unk | Unk | Unk | c.736G>T |
| Kasid | USA | 53 | F | Unk | 8 | 298 | 5 | No | No | No | c.837_838del |
| Holleboom | Holland | 49 | M | Unk | 25 | 118 | 73 | Unk | Unk | Unk | c.964C>T |
| Holleboom | Holland | 54 | F | Unk | 22 | 85 | 56 | Unk | Unk | Unk | c.1012C>T, c.1039C>T |
| Calabresi | Italy | Unk | Unk | Unk | Unk | Unk | Unk | Unk | Unk | Unk | c.1021G>A |
| Cohen | USA | Unk | Unk | Unk | Unk | Unk | Unk | Unk | Unk | Unk | c.1103G>T |
| Calabresi | Italy | Unk | Unk | Unk | Unk | Unk | Unk | Unk | Unk | Unk | c.1115-1117del |
| Calabresi | Italy | Unk | Unk | Unk | Unk | Unk | Unk | Unk | Unk | Unk | c.1234G>A |
| Calabresi | Italy | Unk | Unk | Unk | Unk | Unk | Unk | Unk | Unk | Unk | c.1289C>T |

Abbreviations: Unk: unknown, N: no, M: male, F: female

**Supplementary table 6:** The following terms were cross-referenced with the keywords and [MeSH] terms for ethnicity:

| 1 | LCAT | 32 | Partial LCAT enzyme deficiency and compound heterozygotes | 63 | Fish-eye disease (FED) and African-Americans |
| --- | --- | --- | --- | --- | --- |
| 2 | Fish-eye disease (FED) | 33 | Partial LCAT enzyme deficiency and corneal opacities | 64 | Fish-eye disease (FED) and Hispanic-Americans |
| 3 | Familial Lecithin cholesterol acyl transferase deficiency (FLD) | 34 | Partial LCAT enzyme deficiency and corneal clouding | 65 | Fish-eye disease (FED) and American native continental ancestry group |
| 4 | Partial LCAT enzyme deficiency | 35 | Partial LCAT enzyme deficiency and low high-density cholesterol levels | 66 | Fish-eye disease (FED) and Ameri-Indian |
| 5 | Total LCAT enzyme deficiency | 36 | Partial LCAT enzyme deficiency and anemia | 67 | Fish-eye disease (FED) and Oceanic ancestry group |
| 6 | LCAT gene mutation and polymorphism | 37 | Partial LCAT enzyme deficiency and renal failure | 68 | Fish-eye disease (FED) and Black |
| 7 | LCAT and homozygotes | 38 | Partial LCAT enzyme deficiency and atherosclerosis | 69 | Fish-eye disease (FED) and Hispanic |
| 8 | LCAT and compound heterozygotes | 39 | Total LCAT enzyme deficiency and homozygotes | 70 | Fish-eye disease (FED) and Latino |
| 9 | LCAT and corneal opacities | 40 | Total LCAT enzyme deficiency and compound heterozygotes | 71 | Fish-eye disease (FED) and Asian American |
| 10 | LCAT and corneal clouding | 41 | Total LCAT enzyme deficiency and corneal opacities | 72 | Fish-eye disease (FED) and African American |
| 11 | LCAT and low high-density cholesterol levels | 42 | Total LCAT enzyme deficiency and corneal clouding | 73 | Fish-eye disease (FED) and Native American |
| 12 | LCAT and anemia | 43 | Total LCAT enzyme deficiency and low high-density cholesterol levels | 74 | Fish-eye disease (FED) and Indian |
| 13 | LCAT and renal failure | 44 | Total LCAT enzyme deficiency and anemia | 75 | Fish-eye disease (FED) and Asian |
| 14 | LCAT and atherosclerosis | 45 | Total LCAT enzyme deficiency and renal failure | 76 | Fish-eye disease (FED) and Pacific islander |
| 15 | Fish-eye disease (FED) and homozygotes | 46 | Total LCAT enzyme deficiency and atherosclerosis | 77 | Familial Lecithin cholesterol acyl transferase deficiency (FLD) and Minority groups |
| 16 | Fish-eye disease (FED) and compound heterozygotes | 47 | LCAT gene mutation and polymorphism and homozygotes | 78 | Familial Lecithin cholesterol acyl transferase deficiency (FLD) and Ethnic groups |
| 17 | Fish-eye disease (FED) and corneal opacities | 48 | LCAT gene mutation and polymorphism and compound heterozygotes | 79 | Familial Lecithin cholesterol acyl transferase deficiency (FLD) and African-Americans |
| 18 | Fish-eye disease (FED) and corneal clouding | 49 | LCAT gene mutation and polymorphism and corneal opacities | 80 | Familial Lecithin cholesterol acyl transferase deficiency (FLD) and Hispanic-Americans |
| 19 | Fish-eye disease (FED) and low high-density cholesterol levels | 50 | LCAT gene mutation and polymorphism and corneal clouding | 81 | Familial Lecithin cholesterol acyl transferase deficiency (FLD) and American native continental ancestry group |
| 20 | Fish-eye disease (FED) and anemia | 51 | LCAT gene mutation and polymorphism and low high-density cholesterol | 82 | Familial Lecithin cholesterol acyl transferase deficiency (FLD) and Ameri-indian |
| 21 | Fish-eye disease (FED) and renal failure | 52 | LCAT gene mutation and polymorphism and anemia | 83 | Familial Lecithin cholesterol acyl transferase deficiency (FLD) and Oceanic ancestry group |
| 22 | Fish-eye disease (FED) and atherosclerosis | 53 | LCAT gene mutation and polymorphism and renal failure | 84 | Familial Lecithin cholesterol acyl transferase deficiency (FLD) and Black |
| 23 | Familial Lecithin cholesterol acyl transferase deficiency (FLD) and homozygotes | 54 | LCAT gene mutation and polymorphism and atherosclerosis | 85 | Familial Lecithin cholesterol acyl transferase deficiency (FLD) and Hispanic |
| 24 | Familial Lecithin cholesterol acyl transferase deficiency (FLD) and compound heterozygotes | 55 | LCAT and ethnicity | 86 | Familial Lecithin cholesterol acyl transferase deficiency (FLD) and Latino |
| 25 | Familial Lecithin cholesterol acyl transferase deficiency (FLD) and corneal opacities | 56 | Fish-eye disease (FED) and ethnicity | 87 | Familial Lecithin cholesterol acyl transferase deficiency (FLD) and Asian American |
| 26 | Familial Lecithin cholesterol acyl transferase deficiency (FLD) and corneal clouding | 57 | Familial Lecithin cholesterol acyl transferase deficiency (FLD) and ethnicity | 88 | Familial Lecithin cholesterol acyl transferase deficiency (FLD) and African american |
| 27 | Familial Lecithin cholesterol acyl transferase deficiency (FLD) and low high-density cholesterol levels | 58 | Partial LCAT enzyme deficiency and ethnicity | 89 | Familial Lecithin cholesterol acyl transferase deficiency (FLD) and Native American |
| 28 | Familial Lecithin cholesterol acyl transferase deficiency (FLD) and anemia | 59 | Total LCAT enzyme deficiency and ethnicity | 90 | Familial Lecithin cholesterol acyl transferase deficiency (FLD) and Indian |
| 29 | Familial Lecithin cholesterol acyl transferase deficiency (FLD) and renal failure | 60 | LCAT gene mutation and polymorphism and ethnicity | 91 | Familial Lecithin cholesterol acyl transferase deficiency (FLD) and Asian |
| 30 | Familial Lecithin cholesterol acyl transferase deficiency (FLD) and atherosclerosis | 61 | Fish-eye disease (FED) and Minority groups | 92 | Familial Lecithin cholesterol acyl transferase deficiency (FLD) and Pacific islander |
| 31 | Partial LCAT enzyme deficiency and homozygotes | 62 | Fish-eye disease (FED) and Ethnic groups |  |  |

**Supplementary table 7: PRISMA-S Checklist**

| **Section/topic** | **#** | **Checklist item** | **Location(s) Reported Page #** |
| --- | --- | --- | --- |
| **INFORMATION SOURCES AND METHODS** | | | |
| Database name | 1 | Name each individual database searched, stating the platform for each. | 6 |
| Multi-database searching | 2 | If databases were searched simultaneously on a single platform, state the name of the platform, listing all of the databases searched. | NA |
| Study registries | 3 | List any study registries searched. | NA |
| Online resources and browsing | 4 | Describe any online or print source purposefully searched or browsed (e.g., tables of contents, print conference proceedings, web sites), and how this was done. | 6 |
| Citation searching | 5 | Indicate whether cited references or citing references were examined, and describe any methods used for locating cited/citing references (e.g., browsing reference lists, using a citation index, setting up email alerts for references citing included studies). | 6 |
| Contacts | 6 | Indicate whether additional studies or data were sought by contacting authors, experts, manufacturers, or others. | NA |
| Other methods | 7 | Describe any additional information sources or search methods used. | 6 |
| SEARCH STRATEGIES | | | |
| Full search strategies | 8 | Include the search strategies for each database and information source, copied and pasted exactly as run. | Supplementary material |
| Limits and restrictions | 9 | Specify that no limits were used, or describe any limits or restrictions applied to a search (e.g., date or time period, language, study design) and provide justification for their use. | 6 |
| Search filters | 10 | Indicate whether published search filters were used (as originally designed or modified), and if so, cite the filter(s) used. | NA |
| Prior work | 11 | Indicate when search strategies from other literature reviews were adapted or reused for a substantive part or all of the search, citing the previous review(s). | NA |
| Updates | 12 | Report the methods used to update the search(es) (e.g., rerunning searches, email alerts). | NA |
| Dates of searches | 13 | For each search strategy, provide the date when the last search occurred. | 6 |
| PEER REVIEW | | | |
| Peer review | 14 | Describe any search peer review process. | 6 |
| MANAGING RECORDS | | | |
| Total Records | 15 | Document the total number of records identified from each database and other information sources. | 9 |
| Deduplication | 16 | Describe the processes and any software used to deduplicate records from multiple database searches and other information sources. | 6 |
|  |  |  |  |
| PRISMA-S: An Extension to the PRISMA Statement for Reporting Literature Searches in Systematic Reviews | | |  |
| Rethlefsen ML, Kirtley S, Waffenschmidt S, Ayala AP, Moher D, Page MJ, Koffel JB, PRISMA-S Group. | | |  |
| Last updated February 27, 2020. | |  |  |

**Supplementary table 8 JBI Critical Appraisal Checklist for case reports**

|  | | | |  |  |  |  |  |  |  |  |  |  |  |  |
| --- | --- | --- | --- | --- | --- | --- | --- | --- | --- | --- | --- | --- | --- | --- | --- |
|  |  |  |  |  |  | **1** | **2** | **3** | **4** | **5** | **6** | **7** | **8** | **Overall appraisal** | **Comments** |
|  | **Reviewer** | **Date** | **Author** | **Year** | **Record Number** | **Were patient’s demographic characteristics clearly described?** | **Was the patient’s history clearly described and presented as a timeline?** | **Was the current clinical condition of the patient on presentation clearly described?** | **Were diagnostic tests or assessment methods and the results clearly described?** | **Was the intervention(s) or treatment procedure(s) clearly described?** | **Was the post-intervention clinical condition clearly described?** | **Were adverse events (harms) or unanticipated events identified and described?** | **Does the case report provide takeaway lessons?** |  |  |
| **21** | AJMR | 06/092021 | Okubo | 1996 | 21 | No | Yes | Yes | Yes | Not applicable | Not applicable | Not applicable | Yes | Include | The patient did not receive treatment. |
| **22** | AJMR | 06/092021 | Bujo | 1991 | 22 | Yes | Yes | Yes | Yes | Not applicable | Not applicable | Not applicable | Yes | Include | The patient did not receive treatment. |
| **23** | AJMR | 06/092021 | Miida | 2004 | 23 | Yes | Yes | Yes | Yes | Not applicable | Not applicable | Not applicable | Yes | Include | The patient did not receive treatment. |
| **24** | AJMR | 06/092021 | Yang | 1997 | 24 | Yes | Yes | Yes | Yes | Not applicable | Not applicable | Not applicable | Yes | Include | The patient did not receive treatment. |
| **25** | AJMR | 06/092021 | Naito | 2013 | 25 | Yes | Yes | Yes | Yes | Yes | Yes | No | Yes | Include | They use an experimental treatment with positive partial results. |
| **26** | AJMR | 06/14/2021 | Katayama | 2011 | 26 | Yes | Yes | Yes | Yes | Not applicable | Not applicable | Not applicable | Yes | Include | The patient did not receive treatment. |
| **27** | AJMR | 06/14/2021 | Gotoda | 1991 | 27 | Yes | Yes | Yes | Yes | Not applicable | Not applicable | Not applicable | Yes | Include | The patient did not receive treatment. |
| **28** | AJMR | 06/14/2021 | Tsuchiya | 2011 | 28 | Yes | Yes | Yes | Yes | Not applicable | Not applicable | Not applicable | Yes | Include | The patient did not receive treatment. |
| **29** | AJMR | 06/14/2021 | Hirashio | 2010 | 29 | Yes | Yes | Yes | Yes | Not applicable | Not applicable | Not applicable | Yes | Include | The patient did not receive treatment. |
| **30** | AJMR | 06/14/2021 | Wang | 2011 | 30 | No | Yes | Yes | Yes | Not applicable | Not applicable | Not applicable | Yes | Include | The patient did not receive treatment. |
| **31** | AJMR | 06/14/2021 | Maruyama | 2004 | 31 | Yes | Yes | Yes | Yes | Not applicable | Not applicable | Not applicable | Yes | Include | This is not a case report, but an original article including analysis of primary hyperlipidemias, including LCAT deficiency. |
| **32** | AJMR | 06/17/2021 | Moriyama | 1995 | 32 | Yes | Yes | Yes | Yes | Not applicable | Not applicable | Not applicable | Yes | Include | The patient did not receive treatment. |
| **33** | AJMR | 06/17/2021 | Maeda | 1991 | 33 | Yes | Yes | Yes | Yes | Not applicable | Not applicable | Not applicable | Yes | Include | The patient did not receive treatment. |
| **34** | AJMR | 06/17/2021 | Kanai | 2018 | 34 | Yes | Yes | Yes | Yes | Not applicable | Not applicable | Not applicable | Yes | Include | The patient did not receive treatment. |
| **35** | AJMR | 06/18/2021 | Park | 2009 | 35 | Yes | Yes | Yes | Yes | Not applicable | Not applicable | Not applicable | Yes | Include | The patient did not receive treatment. |
| **36** | AJMR | 06/18/2021 | Holleboom | 2011 | 36 | Yes | Yes | Yes | Yes | Not applicable | Not applicable | Not applicable | Yes | Include | The patient did not receive treatment. |
| **37** | AJMR | 06/18/2021 | Contacos | 1996 | 37 | Yes | Yes | Yes | Yes | Not applicable | Not applicable | Not applicable | Yes | Include | The patient did not receive treatment. |
| **38** | AJMR | 06/18/2021 | Hörl | 2006 | 38 | Yes | Yes | Yes | Yes | Not applicable | Not applicable | Not applicable | Yes | Include | The patient did not receive treatment. |
| **39** | AJMR | 06/18/2021 | Steyrer | 1995 | 39 | Yes | Yes | Yes | Yes | Not applicable | Not applicable | Not applicable | Yes | Include | The patient did not receive treatment. |
| **40** | AJMR | 06/18/2021 | Wiebusch | 1995 | 40 | Yes | Yes | Yes | Yes | Not applicable | Not applicable | Not applicable | Yes | Include | The patient did not receive treatment. |
| **41** | AJMR | 06/18/2021 | Winder | 1999 | 41 | Yes | Yes | Yes | Yes | Not applicable | Not applicable | Not applicable | Yes | Include | The patient did not receive treatment. |
| **42** | AJMR | 06/18/2021 | Rader | 1994 | 42 | Yes | Yes | Yes | Yes | Not applicable | Not applicable | Not applicable | Yes | Include | The patient did not receive treatment. |
| **43** | AJMR | 06/25/2021 | Funke | 1993 | 43 | Yes | Yes | Yes | Yes | Not applicable | Not applicable | Not applicable | Yes | Include | The patient did not receive treatment. |
| **44** | AJMR | 06/25/2021 | Weber | 2007 | 44 | Yes | Yes | Yes | Yes | Not applicable | Not applicable | Not applicable | Yes | Include | The patient did not receive treatment. |
| **45** | AJMR | 06/25/2021 | Nanjee | 2003 | 45 | Yes | Yes | Yes | Yes | Not applicable | Not applicable | Not applicable | Yes | Include | The patient did not receive treatment. |
| **46** | AJMR | 06/25/2021 | Argyropoulos | 1998 | 46 | Yes | Yes | Yes | Yes | Not applicable | Not applicable | Not applicable | Yes | Include | The patient did not receive treatment. |
| **47** | AJMR | 06/25/2021 | Kuivenhoven | 1996 | 47 | Yes | Yes | Yes | Yes | Not applicable | Not applicable | Not applicable | Yes | Include | The patient did not receive treatment. |
| **48** | AJMR | 06/25/2021 | Holleboom | 2011 | 48 | No | Yes | Yes | Yes | Not applicable | Not applicable | Not applicable | Yes | Include | The patient did not receive treatment. |
| **49** | AJMR | 06/26/2021 | Kuivenhoven | 1996 | 49 | Yes | Yes | Yes | Yes | Not applicable | Not applicable | Not applicable | Yes | Include | The patient did not receive treatment. |
| **50** | AJMR | 06/26/2021 | Kuivenhoven | 1997 | 50 | Not applicable | Yes | Yes | Yes | Not applicable | Not applicable | Not applicable | Yes | Include | This article presents an overview of LCAT deficiency syndromes until that year. |
| **51** | AJMR | 06/26/2021 | Kuivenhoven | 1995 | 51 | Yes | Yes | Yes | Yes | Not applicable | Not applicable | Not applicable | Yes | Include | The patient did not receive treatment. |
| **52** | AJMR | 06/26/2021 | Miettinen | 1997 | 52 | Yes | Yes | Yes | Yes | Not applicable | Not applicable | Not applicable | Yes | Include | The patient did not receive treatment. |
| **53** | AJMR | 06/26/2021 | Gylling | 1992 | 53 | Yes | Yes | Yes | Yes | Not applicable | Not applicable | Not applicable | Yes | Include | The patient did not receive treatment. |
| **54** | AJMR | 06/26/2021 | Miettinen | 1994 | 54 | Yes | Yes | Yes | Yes | Not applicable | Not applicable | Not applicable | Yes | Include | The patient did not receive treatment. |
| **55** | AJMR | 06/26/2021 | Teh | 1999 | 55 | Yes | Yes | Yes | Yes | Not applicable | Not applicable | Not applicable | Yes | Include | The patient did not receive treatment. |
| **56** | AJMR | 06/26/2021 | Klein | 1993 | 56 | Yes | Yes | Yes | Yes | Not applicable | Not applicable | Not applicable | Yes | Include | The patient did not receive treatment. |
| **57** | AJMR | 06/28/2021 | Guerin | 1997 | 57 | Yes | Yes | Yes | Yes | Not applicable | Not applicable | Not applicable | Yes | Include | The patient did not receive treatment. |
| **58** | AJMR | 06/28/2021 | Elkhalil | 1997 | 58 | Yes | Yes | Yes | Yes | Not applicable | Not applicable | Not applicable | Yes | Include | The patient did not receive treatment. |
| **59** | AJMR | 06/28/2021 | Dorval | 1993 | 59 | No | No | Yes | Yes | Not applicable | Not applicable | Not applicable | Yes | Include | The patient did not receive treatment. |
| **60** | AJMR | 06/28/2021 | Baass | 2009 | 60 | Yes | Yes | Yes | Yes | Not applicable | Not applicable | Not applicable | Yes | Include | The patient did not receive treatment. |
| **61** | AJMR | 06/28/2021 | Funke | 1991 | 61 | Yes | Yes | Yes | Yes | Not applicable | Not applicable | Not applicable | Yes | Include | The patient did not receive treatment. |
| **62** | DBEL | 22/06/21 | Hanns-Georg Klein | 1991 | 62 | Yes | Yes | Yes | Yes | Not applicable | Not applicable | Yes | Yes | Include | The patient did not receive treatment. |
| **63** | DBEL | 22/06/21 | Bernhard U. Bender | 2007 | 63 | Yes | Yes | Yes | Yes | Yes | Yes | Not applicable | Yes | Include | The patient did not receive treatment. |
| **64** | DBEL | 22/06/21 | James S. Owen | 1996 | 64 | Yes | Yes | Yes | Yes | Not applicable | Not applicable | Not applicable | Yes | Include | The patient did not receive treatment. |
| **65** | DBEL | 22/06/21 | Fountoulakis | 2019 | 65 | Yes | Yes | Yes | Yes | Yes | Yes | Yes | Yes | Include | The article is a large cohort. |
| **66** | DBEL | 22/06/21 | Calabresi | 2005 | 66 | No | Yes | Yes | Yes | Not applicable | Not applicable | Not applicable | Yes | Include | The article is a large cohort. |
| **67** | DBEL | 22/06/21 | Calabresi | 2009 | 67 | No | Yes | Yes | Yes | Not applicable | Not applicable | Not applicable | Yes | Include | The article is a large cohort. |
| **68** | DBEL | 22/06/21 | Gigante | 2006 | 68 | Yes | Yes | Yes | Yes | Not applicable | Not applicable | Not applicable | Yes | Include | The patient did not receive treatment. |
| **69** | DBEL | 22/06/21 | Taramelli | 1990 | 69 | Yes | Yes | Yes | Yes | Not applicable | Not applicable | Not applicable | Yes | Include | The patient did not receive treatment. |
| **70** | DBEL | 22/06/21 | Frasca | 2004 | 70 | Yes | Yes | Yes | Yes | Not applicable | Not applicable | Not applicable | Yes | Include | The patient did not receive treatment. |
| **71** | DBEL | 22/06/21 | Sessa | 2001 | 71 | Yes | Yes | Yes | Yes | Not applicable | Not applicable | Not applicable | Yes | Include | The patient did not receive treatment. |
| **72** | DBEL | 23/06/21 | Conca | 2012 | 72 | Yes | Yes | Yes | Yes | Not applicable | Not applicable | Not applicable | Yes | Include | The patient did not receive treatment. |
| **73** | DBEL | 23/06/21 | Aranda | 2008 | 73 | Yes | Yes | Yes | Yes | Yes | Yes | Yes | Yes | Include |  |
| **74** | DBEL | 23/06/21 | Skretting | 1992 | 74 | Yes | Yes | Yes | Yes | Not applicable | Not applicable | Not applicable | Yes | Include | The patient did not receive treatment. |
| **75** | DBEL | 23/06/21 | Strøm | 2011 | 75 | Yes | Yes | Yes | Yes | Yes | Yes | Yes | Yes | Include | The patient did not receive treatment. |
| **76** | DBEL | 23/06/21 | Idzior-Walus | 2006 | 76 | Yes | Yes | Yes | Yes | Yes | No | No | Yes | Include |  |
| **77** | DBEL | 23/06/21 | Castro-Ferreira | 2017 | 77 | Yes | Yes | Yes | Yes | Yes | Yes | Yes | Yes | Include | The patient did not receive treatment. |
| **78** | DBEL | 23/06/21 | Rial-Crestelo | 2017 | 78 | Yes | Yes | Yes | Yes | Not applicable | Not applicable | Not applicable | Yes | Include | The patient did not receive treatment. |
| **79** | DBEL | 23/06/21 | Cirera | 1998 | 79 | Yes | Yes | Yes | Yes | Not applicable | Not applicable | Not applicable | Yes | Include | The patient did not receive treatment. |
| **80** | DBEL | 23/06/21 | Recalde | 2002 | 80 | Not applicable | Not applicable | Not applicable | Not applicable | Not applicable | Not applicable | Not applicable | Yes | Include | The article is a review. |
| **81** | DBEL | 23/06/21 | Blanco-Vaca | 1997 | 81 | Yes | Yes | Yes | Yes | Not applicable | Not applicable | Not applicable | Yes | Include | The patient did not receive treatment. |
| **82** | DBEL | 24/06/21 | Lamiquiz-Moneo | 2019 | 82 | Yes | Yes | Yes | Yes | Yes | Yes | Yes | Yes | Include | The patient did not receive treatment. |
| **83** | DBEL | 24/06/21 | Skretting | 1992 | 83 | Yes | Yes | Yes | Yes | Not applicable | Not applicable | Not applicable | Yes | Include | The patient did not receive treatment. |
| **84** | DBEL | 24/06/21 | Brites | 1998 | 84 | Yes | Yes | Yes | Yes | Yes | Yes | Yes | Yes | Include |  |
| **85** | DBEL | 24/06/21 | Norum | 2020 | 85 | Not applicable | Not applicable | Not applicable | Not applicable | Not applicable | Not applicable | Not applicable | Yes | Include | The article is a review. |
| **86** | DBEL | 24/06/21 | Sampaio | 2017 | 86 | No | No | No | No | No | No | No | Yes | Include | The article is a large cohort. |
| **87** | DBEL | 24/06/21 | Tobar | 2019 | 87 | Yes | Yes | Yes | Yes | Not applicable | Not applicable | Not applicable | Yes | Include | The patient did not receive treatment. |
| **88** | DBEL | 24/06/21 | Marin | 2006 | 88 | Yes | Yes | Yes | Yes | Yes | Yes | Yes | Yes | Include | The patient did not receive treatment. |
| **89** | DBEL | 24/06/21 | Palmiero | 2009 | 89 | Yes | Yes | Yes | Yes | Not applicable | Not applicable | Not applicable | Yes | Include | The patient did not receive treatment. |
| **90** | DBEL | 24/06/21 | Posadas | 2014 | 90 | Yes | Yes | Yes | Yes | Not applicable | Not applicable | Not applicable | Yes | Include | The patient did not receive treatment. |
| **91** | DBEL | 24/06/21 | Mahapatra | 2015 | 91 | Yes | Yes | Yes | Yes | Yes | Yes | Yes | Yes | Include |  |
| **92** | DBEL | 24/06/21 | Muthusethupathi | 1999 | 92 | Yes | Yes | Yes | Yes | Yes | Yes | Yes | Yes | Include | The pacient recived a renal transplant. |
| **93** | DBEL | 25/06/21 | Gopalakrishnan | 2016 | 93 | Yes | Yes | Yes | Yes | Not applicable | Not applicable | Not applicable | Yes | Include | The pacient did not recive treatment. |
| **94** | DBEL | 25/06/21 | Oliaei | 2019 | 94 | No | No | No | No | No | No | No | Yes | Include | The article is a large cohort. |
| **95** | DBEL | 25/06/21 | Roshan | 2011 | 95 | Yes | Yes | Yes | Yes | Yes | Yes | Yes | Yes | Include |  |
| **96** | DBEL | 25/06/21 | Charlton-Menys | 2007 | 96 | Yes | Yes | Yes | Yes | Not applicable | Not applicable | Not applicable | Yes | Include | The patient did not receive treatment. |
| **97** | DBEL | 25/06/21 | Ustaoglu | 2019 | 97 | Yes | Yes | Yes | Yes | Not applicable | Not applicable | Not applicable | Yes | Include | The patient did not receive treatment. |
| **98** | DBEL | 25/06/21 | Dimick | 2014 | 98 | Yes | Yes | Yes | Yes | Yes | Yes | Yes | Yes | Include |  |
| **99** | DBEL | 25/06/21 | Kasid | 2001 | 99 | Yes | Yes | Yes | Yes | Not applicable | Not applicable | Not applicable | Yes | Include | The patient did not receive treatment. |
| **100** | DBEL | 25/06/21 | Cohen | 2013 | 100 | No | No | No | No | No | No | No | Yes | Include | The article is a review. |
| **101** | DBEL | 25/06/21 | Klein | 1993 | 101 | Yes | Yes | Yes | Yes | Not applicable | Not applicable | Not applicable | Yes | Include | The patient did not receive treatment. |
| **102** | DBEL | 25/06/21 | Miller | 1995 | 102 | Yes | Yes | Yes | Yes | Not applicable | Not applicable | Not applicable | Yes | Include | The patient did not receive treatment. |
